# Water the Source of Malarial Poison

**Published:** 1897-06

**Authors:** J. E. Gardiner

**Affiliations:** Medical Sanitary Inspector for Equitable Life Assurance Society


For the Texas Medical Journal.

### WATER THE SOURCE OF MALARIAL POISON.

BY J. E. GARDINER, M. D.,
Medical Sanitary Inspector for Equitable Life Assurance Society.

I WISH to bring to the notice of the medical profession, as well as that of the laity, some very interesting data demonstrating the water source of malarial poison.

The term malaria—though a misnoma—has long been used empirically to designate a specific type of disease, usually, quite easily differentiated from all other forms of ailment. The consensus of medical opinion *may* still favor the *theory* of *aerial* dissemination of the specific materis morbi, generating malarial fevers and other characteristic organic derangement common to moist heat and decaying vegetation in low lying districts. I had always been an enthusiastic advocate of the theory of atmospheric infection of this common trouble, until about one year ago, when such strong proofs were brought to our notice that water constituted the medium of malarial poison, that I resolved to solve the perplexing problem by further scientific research and daily observation. Having been at about this time engaged by the Equitable Life Assurance Society of New York, as special medical sanitary inspector for the States of Texas and Arkansas and the Territories of Indian and Oklahoma. I possessed a fair and rare opportunity of procuring and accumulating statistics, pro and con, bearing upon this very important subject.

The territory thus forming the area of my observation and inquiry, is about 800 miles square, or 64,000 square miles, and no doubt contains as great a variety of soil and climate as that amount of space anywhere else in the world. From the massive accumulation of primæ facie evidence, from daily personal research, and the innumerable data furnished me by many of the mort prominent members of medical profession, as well as by intelligent citizens of other vocations—all of whom residing in the most malarious sections of the above described area—that I am now forcibly constrained to accept an adverse doctrine to that of the aerial propagation of the malarial bacilli.

I feel justified in the belief that the results of scientific research made in this section, will prove to be as convincing and conclusively acceptable as that obtained from any other field of observation.

The theories of the contaminated atmosphere being the medium of endemic and malarial diseases are without logical or scientific support, and are as visionary as describing a shadow for its substance. All advocates of the aerial theory of malaria, admit that the special toxine is generated on or in the earth's surface, in time of heat and moisture; and is evolved from its nidus by evaporations, and is low-lying—wafted and disseminated by the atmosphere. Water, therefore, contained in and on the earth's surface must contain the same or a like ratio of malarial poison to that of the air; that the amount of vaporized water in the air bears to the body of surface water from which the evaporation proceeded; and if the air we breath contains, to such an inimical degree, this poison, as to produce the multitudinous ills resulting from its inhalation and absorption by man, that we are habitually ascribing to it, then water being its primal, and drank in the usual quantity per diem would proportionately be more disastrous in its deadly effects. There are tangible evidences within the scope of nearly every physicians' daily practice, which points as unerringly to the water source of malaria, as the mariner's needle indicates the north pole. But many of the brilliant professional lights to-day repose with pleased and manifest credence in the long accepted aerial theory of malarial plasmodium, and vehemently controvert the introduction of any innovating dogma. Theories, however, are at best, but logical guesses, and are to the scientist what the exploring shaft is to the miner, or a trail to the huntsman,—only a blazed way to the goal. And, as in this instance, possessing the goal—truth, we need no longer the trail or theory.

I shall not pretend to point out or touch upon the specific toxine, or micro-organism of malaria, the isolation and description of which belongs more suitably to the domain of the bacteriologist and microscopist in his laboratory.

I earnestly wish, however, by the following unique data, obtained within the past twelve months, to clearly and convincingly demonstrate, that the plasmodium malariæ is a resident of water, and through that medium finds ingress to the human system. During last summer and fall, notably the most seasonable periods of the year for malarial fevers, I inspected the sanitary conditions of the Indian and Oklahoma Territories and the State of Arkansas—and in the body of my report upon the topography and hygiene of the latter, I say, in part, that on account of the low swampy condition, flat surface, inferior drainage, numerous lagoons, the many slow and sluggish streams, luxuriant vegetation with intense heat and the constant moist atmosphere from evaporations and frequent rains, this section has been stigmatized as one of the most deadly malarious parts of the United States. Because, up to a few years ago, citizens procured drinking water only from open, shallow wells, creeks and rivers, typhoid and all the forms of malarial fevers, of the most pernicious types, prevailed persistently throughout the year, but cases were more numerous and fatal during the hot seasons, sweeping through the country, decimating its inhabitants like a veritable oriental plague.

The insalubrity of this section has been the greatest impedimediment to its development and progress. Though now the light of truth is rapidly dawning upon this people, and the owners of saw-mills, wood factories and other large property owners located in the most densely malarious districts, are substituting with most marvelous and salubrious effect—cistern, artersian and sterilized water, for the former surface water used for domestic purposes. Statistics showed that in many places, cistern water alone, had lessened mortality rate 500 per cent. For instance, Mr. Foster, who furnished the following data, owned and operated a large saw mill in La Fayette county, who employed 120 laborers, out of which number six had died from malarial troubles in 1895, while using surface water from wells and creeks. In 1896, the same number of men employed at the same mill, and drinking only cistern water which had been supplied by the owner of the mill, and without any other hygienic improvement, passed the entire year with peculiar, though perfect immunity from all the inherent troubles of a malarial origin.

While in Mariana, Ark., I visited the large saw mill of Mr. Miller, on the bank of St. Francis river. Low swampy lands, reeking with luxuriant and decomposing vegetation surround this little town, and ague is the bane of this fertile section. For two years previous to that of 1896, the engineers informed me all the employes of the mill drank only well and river water, followed in each case with the usual sequence of the inevitable chills and fevers. During last year, four of these same employes, the engineer being one of the number, drank only distilled water from the engine, cooled with ice. None of these men had any malarial trouble during the entire year, except the engineer himself, who had two slight fevers, which followed successively in a few days, the ingestion of some impure water, at as many different times. All of the other employes of the mill drinking water from the same source, though unboiled, were sickened as formerly, with the usual malarial attacks, just as the entire force had been previous to the adoption of the sterilized water, by those four above mentioned. One laborer at this place was brought in from the swamps in a low state of swamp fever, was put under the best medical care, and would improve slightly for a short time, and relapse as often, and naturally growing weaker all the while, finally he abandoned all medical treatment, and began to drink the distilled ice water at the mill, in lieu of the surface water which he had been drinking up to this time. He at once began rapidly to improve, and was soon a well and hearty man, and this without any other change in habits or hygience than non-malarious water. Up to about twelve years ago, Fulton, Ark., was reported to have been one of the most deadly malarious and pestilential towns in that State. Every citizen able to leave during the hot and most unhealthy seasons, would do so to escape the severe outbreaks of malarial fevers. Shallow wells, creeks and the river, supplied exclusively, all the drinking water, not only for the town, but for all the surrounding country, which shared a like fate. About this time artesian water was procured for the town, and as the people adopted its use for drinking and culinary purposes, the place became one of the most salubrious in the State, and this too without the perceptible aid of any other hygienic measure. The towns of Helena and Forest City, Ark., furnish most striking proofs of the water source of malaria. Helena has artesian, and Forest City very deep well water supplies for general purposes, and the leading physicians and citizens of both places informed me that the general health of the respective towns had improved with the supply of a superior quality of water alone, at least 50 per cent.

Every city, town and community, whether in the mountainous or swampy regions of the State furnished the same convincing proof of the water, and not the air, being the medium of malarial poison. The introduction of bored and drilled wells in the mountainous districts of Arkansas, which act as recepticles for surface drainage on the slopes of hills and in the valleys where they are located, are coincident with a low grade of remittent fever—evidently of malarial origin confined to such of the people as drink water from this source. The following personal experience of a prominent physician at Clarendon, Ark., was given me last December, by the gentleman in person. For seven years he and his wife and several children have resided in that malarious town, located on the bank of White River, just north of, and bordering on one of the most uninhabitable and impenetrable swampy districts in the South. During the entire period he and his children have drank no other than boiled water, and all have been especially free from all forms of malaria, while the wife, who confining herself to the exclusive use of well water, refused to drink sterilized water for the first three years of the seven, claiming it to be insipid and intolerable, was in consequence, a constant victim of malarial toxemia. Her depreciated health finally gave rise to serious apprehensions for her physical well being, and seeing her husband and children enjoying constantly such perfect health, decided to forego her antipathy to boiled water, and give it a trial. Rapid and complete restoration of her health succeeded the experiment, and perfect immunity from her former complaints has been the continued blessing of her persistent use of boiled drinking water during the last four years, in common with the other members of her family. The adoption of a pure, absolutely non-malarious water can alone be credited for these unparalleled phenomena.

One of the first striking instances attracting my attention to malarious water was last May in the Indian Territory—in a dirty-looking, uninviting little village on the broad and fertile alluvial land of the Arkansaw river, in the Choctaw Nation. The ill repute of the place, from recent unfavorable reports to the Medical Department of Insurance, had induced our company to put this town and community under the ban—until further inspection. I was delegated for the purpose—and found that the inhabitants in general suffered from the severest types of malarial and typhoid fevers, hematuria, dysentery, etc. All of those so affected were consumers exclusively of shallow well and surface water. Those able to carry, and desiring insurance, were a few merchants, whose drinking water supply was furnished from deep wells sunk into gravel beds below an impervious stratum of clay, and so curbed as to perfectly exclude all surface water. These citizens—numbering some half dozen—and their families had been residing there five years, *drinking only* this deep water, and enjoying *good health* and *perfect* immunity from *every phase* of *malarial disease.* There was positively no other difference in the sanitary environments of these families and their neighbors than the quality of drinking water.

Mr. Stuart, cashier of a bank at Jacksboro, Texas, gave me the following instance of his own experience a few years ago: He owned and resided on a plantation located between two large creeks—the land gracefully inclined from center in either direction towards the creeks—affording magnificent drainage. These creeks in the warm, dry seasons would cease to flow, but maintaining stagnant pools along their beds. The supply of drinking water was furnished from a well whose depth corresponded nearly with the bottom of the creeks, and during the low stage of water of the creeks his family and all who drank water from that well suffered most constantly from malarial fevers. He sank an artesian well, which supplied water for drinking purposes, and with this change of water every vestige of malaria disappeared immediately and permanently. His successors on the plantation ever since have experienced the same happy immunity from malaria while drinking artesian water. The community yet continue to be victims of malaria, being still supplied with and using only surface water. No auxiliary sanitary measures were inaugurated to deprive the pure quality of water of this healthful change.

On the banks of Red River, near Jefferson, Texas, is a small farm of about one hundred acres, very fertile, producing yearly two bales of cotton per acre, and owned by Mr. Freeman, wholesale merchant at Jefferson, who furnished me with the following remarkable data: After the farm had been cultivated unsuccessfully for several years, on account of the most deadly forms of malarial fevers among the tenants the farm was abandoned for two years because of its unsalubrity. At the end of this time, Mr. F., trying an experiment to redeem it, constructed a new cottage and built a cistern to supply drinking water for the manager and his family. Notwithstanding the abundant yield of produce to the tiller not one could be induced to go on the place until the owner pledged himself to assume all medical bills for the current year for a good farmer and his family, and that after any length of time during the year he could not remain, because of malaria, he could remove and live in town the remainder of the year at his—Mr. F.'s expense—provided the tenant, while on the property, confined himself and family to the exclusive use of cistern water, which agreement was complied with, and not a case of malaria was experienced by any of the family the entire year. The radical change in health of this alluvial and luxuriant spot during the last eight years has induced scores of reliable farmers to apply for the control of the plantation, who could not be influenced before through mercenary motives to go there.

Dr. Chas. Smart, major and surgeon, U. S. A., in a masterly article contributed to the reports and papers of the American public Health Association, 1892, Vol. xviij, page 307, cites some of the most convincing proofs of water causation of malaria. He gives an instance at Fort Bridges, Wyoming, where the post was situated at an altitude of 7000 feet; its maximum temperature of its hottest months was 87° F.; its mean, 65° F. No marshes were to be found in its neighborhood. The streams which passed through it were bold and rapid, with high banks. But that he found always, upon "investigating seasonable differences in the water supply, that the impurities coincided with the prevalence of remittent fevers." "Troops from Florida imbued with ague would rapidly recover their health and spirits, and it was certainly a non-malarious station, and yet even when the organic constituents of the stream which supplied the drinking water were such as to yield high rates of albuminoid ammonia, remittent fevers became prevalent." Many other facts, he goes on to say, entered into the argument to prove the causation of these fevers by the water supply. These experiences were some twenty years ago.

Further on in the same article the writer goes on to say that Fort Brown, Texas, must be set down as the most unhealthy post in the *country*; that the garrison averaged nearly two attacks yearly for each man, which made 1986.11 per thousand admissions, according to report for 1889; and again, in 1890, Fort Brown is shown to have the worst record of any post in the army. In 1891 a great change took place. Instead of 1876 admissions per thousand of strength it had only 325.91, and during the last year a rapid improvement in the health of the post was observed, the rate of admissions for malarial fevers being only 16.13 per thousand of strength at this fort, and that of the army having been 62.23. So this post, notwithstanding the uneniable reputation of being the most unhealthy in the army, has changed positions on the list—with at the same time a continuance of all its unsanitary surroundings—the *very worst that could be imagined.* He says, "we had not long to seek for the change at Fort Brown. During the years of unsalubrity the garrison drank the muddy and impure water of the Rio Grande, and during the past and part of the previous year, they drank a non-malarial water. In 1890 an ice machine was sent to the post to furnish the fevered sick at the hospital with ice for cooling draughts. It did not only answer that purpose efficiently, but prevented the very fevers which it had been sent to alleviate—a condensing coil having been attached to the machine to furnish the garrison with cool distilled water, and as it was so much superior to the former supply, it was immediately used by all for drinking and cooking." Surgeon-General Southerland was sufficiently convinced from the above data to recommend the use of ice machines for cooling and distilling water at all other posts, when the water supply was of doubtful quality. This entire article is fairly bristling with the most unique and convincing evidence of water causation of malaria that can possibly be adduced or desired, and I recommend that every one who can procure it should read and re-read it.

The records of the construction of the Y. & M. V. R. R.—running parallel with the Mississippi River—frow New Orleans to Memphis, traversing those marshy and malarious bottoms, furnish another indestructible link in the chain of evidence. The laborers, while drinking water from wells sunk in the mud and soft earth, were so stricken down with malarial fevers that the contractors were about forced to abandon the enterprise, but by the advice of the physician they substituted pure drinking water, which was followed by a rapid recuperation of health and discontinuance of malarial fevers, and the work progressed without any further disturbance.

Some of the farms of the Brazos river bottoms are supplied with artesian water for the tenants by mains conducting the water to the different tenant houses, and as a result of its exclusive use for drinking purposes, malarial fevers have totally disappeared from all, both white and black, who have thus appropriated it. I could continue indefinitely in this manner citing undisputable proofs of the water causation of malaria, but have already taken a vast amount of valuable space, and will content myself by referring to a few prominent places, where prima facie evidence in addition to the foregoing can easily and abundantly be obtained in some of the hitherto most malarious districts in this State, viz: Houston, Waco, Belton, Yoakum, Wharton and many others. On the other hand, point out any town or community without pure drinking water, be it in the marshy and luxuriant bottoms, or in the salubrious and picturesque valleys of the mountain districts, and you can find numerous victims daily prostrate by this destructive and insidious human enemy. Boerne and Kerville are too fitting examples—the former at the foothills in Western Texas, at an altitude of about 1,200 feet, and the latter 40 miles further north, located in a beautiful valley on the banks of a charming mountain stream, at a height of 1640 feet. This is without doubt the most salubrious section in the south, and yet these noted health resorts are visited annually with great numbers of the well known and prevailing type of slow fever.

The intuition or innate sagacity of the much-abused Chinese surpasses by far the boasted wisdom of many of our most advanced and learned teachers. The Chinese laws compel its citizens to boil all their drinking water, and I am reliably informed that those who migrate to this country scrupulously adhere to the same custom wherever they are located. Hence the extensive habit of tea-drinking with them and the phenomenal immunity of this race of people from all forms of malaria, while all other nationalities, born or imported here, are equally susceptible to its baneful effects.

Hoping that, if this paper should fail to convince the skeptical, it will at least inspire him with a desire to make personal observations and research, and the results of which I feel amply certain in predicting, will be practically, consonant with the above data.

